# Space-Time Clustering Characteristics of Tuberculosis in Khyber Pakhtunkhwa Province, Pakistan, 2015–2019

**DOI:** 10.3390/ijerph17041413

**Published:** 2020-02-21

**Authors:** Sami Ullah, Hanita Daud, Sarat C. Dass, Hadi Fanaee-T, Husnul Kausarian, Alamgir

**Affiliations:** 1 Department of Fundamental & Applied Sciences, Universiti Teknologi PETRONAS, Seri Iskandar 32610, Perak, Malaysia; hanita_daud@utp.edu.my; 2 School of Mathematical and Computer Sciences, Heriot-Watt University Malaysia, Putrajaya 62200, Malaysia; s.dass@hw.ac.uk; 3 Center for Applied Intelligent Systems Research (CAISR), Halmstad University, SE-301 18 Halmstad, Sweden; h.f.tork@medisin.uio.no; 4 Department of Geological Engineering, Universitas Islam Riau, Pekanbaru 28284, Indonesia; husnulkausarian@eng.uir.ac.id; 5 Department of Statistics, University of Peshawar, Peshawar 25120, Pakistan; profalamgir@yahoo.com

**Keywords:** tuberculosis, space-time clusters, Khyber Pakhtunkhwa, Pakistan

## Abstract

The number of tuberculosis (TB) cases in Pakistan ranks fifth in the world. The National TB Control Program (NTP) has recently reported more than 462,920 TB patients in Khyber Pakhtunkhwa province, Pakistan from 2002 to 2017. This study aims to identify spatial and space-time clusters of TB cases in Khyber Pakhtunkhwa province Pakistan during 2015–2019 to design effective interventions. The spatial and space-time cluster analyses were conducted at the district-level based on the reported TB cases from January 2015 to April 2019 using space-time scan statistics (SaTScan). The most likely spatial and space-time clusters were detected in the northern rural part of the province. Additionally, two districts in the west were detected as the secondary space-time clusters. The most likely space-time cluster shows a tendency of spread toward the neighboring districts in the central part, and the most likely spatial cluster shows a tendency of spread toward the neighboring districts in the south. Most of the space-time clusters were detected at the start of the study period 2015–2016. The potential TB clusters in the remote rural part might be associated to the dry–cool climate and lack of access to the healthcare centers in the remote areas.

## 1. Introduction

Tuberculosis (TB) is an aerosol transmittable disease that is caused by a bacillus mycobacterium and is mainly spread from a TB-infected person to other nearby people through coughing, sneezing, spitting, or talking. TB is still one of the major public health challenges at the global level, particularly in developing countries, and is listed in the top-ten major causes of mortality at the world-level. It is the second fatal disease After HIV/AIDS caused by a single infectious agent [1,2]. The developing countries such as Nigeria, Philippines, Indonesia, Pakistan, India, South Africa, and China highly contribute (above 60%) to total TB burden in the world, where the diagnosis and treatment of TB are difficult to access [3,4].

Pakistan is one of those developing countries where TB is a major public health challenge. Approximately half a million new TB cases are reported each year, and approximately 70,000 people die each year due to TB disease [5]. In terms of TB case burden, Pakistan ranks 5th in the world due to the high occurrence of multidrug-resistant TB (MDR-TB) cases [3,6,7]. Khyber Pakhtunkhwa is the northwestern province of Pakistan, where approximately 55,000 new cases of TB (all types) are reported each year [5]. During 2002–2017, more than 462,920 TB cases were reported in Khyber Pakhtunkhwa province [8]. 

Several epidemiological studies on TB have been previously conducted in different districts of Khyber Pakhtunkhwa [9,10,11,12,13,14,15,16,17]. These studies focused on the epidemiological characteristics and risk factors analysis of TB within a specific district. However, the space-time cluster analysis of TB incidence at the district-level in the province seems to be lacking. Space-time cluster detection can identify important space-time patterns in TB incidence in Khyber Pakhtunkhwa province, which could be useful for the evidence-based public health interventions to control the TB outbreak [18]. This study aims to identify the space-time clusters of TB cases at the district-level in Khyber Pakhtunkhwa province, Pakistan from January 2015 to April 2019. 

A number of methods have been developed for the automatic detection of disease clusters in public health data. Generally, cluster detection methods are classified as either global or local tests. Global clustering methods are used to assess whether a global tendency for the disease to group together is apparent throughout the study region, but it does not identify the location of clusters [19,20,21,22]. These types of methods are appropriate, for example, for finding evidence of whether a disease is infectious or not. Local methods infer the locations and extent of clusters [23,24,25]. This group of methods (which infer the geographical location and time-period of the clusters) can further be divided into two categories [26]: clustering-based methods [27,28,29] and scan statistics methods [23,24,25]. Scan statistics methods are the most widely used methods in public health and epidemiology [30,31,32,33,34,35,36,37]. 

We used the scan statistics method [23,24,38] to detect the potential space-time clusters of TB cases in Khyber Pakhtunkhwa province, Pakistan. Detecting such clusters helps in identifying the important patterns in the TB incidence, and can thereby provide important information for public health managers to identify their targets of interest for interventions. Moreover, detecting space-time clusters of TB cases assist the epidemiologists in finding the possible environmental or social determinants of TB outbreak in the study area.

## 2. Materials and Methods

### 2.1. Study Area

**Figure 1:**
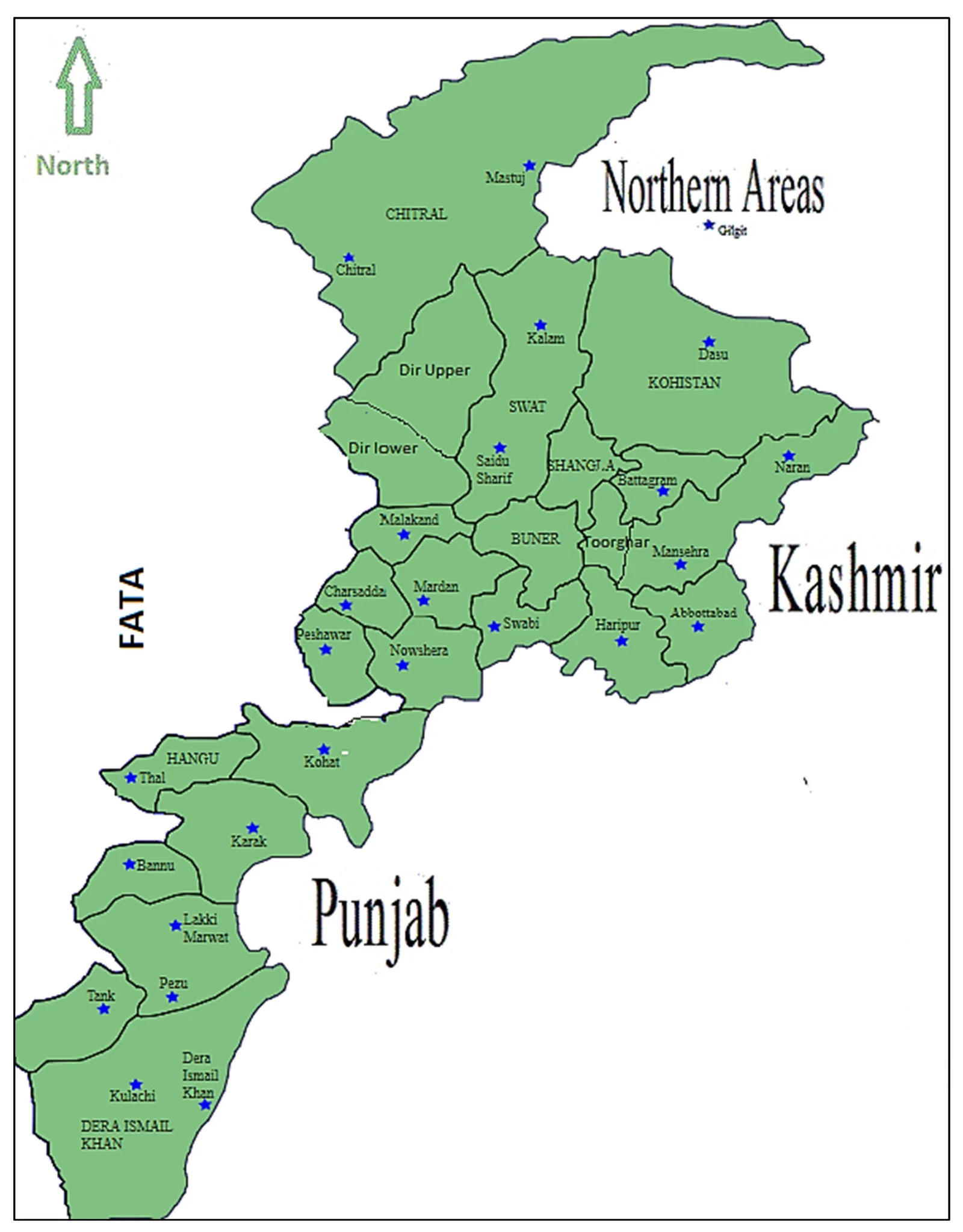
Map of Khyber Pakhtunkhwa province.

The study was carried out in Khyber Pakhtunkhwa, the northwestern province of Pakistan. The total area of Khyber Pakhtunkhwa province is 74,521 km^2^ with a census population of 30.52 million according to the census 2017. The land area of the province has 25 districts of different sizes as shown in Figure 1. However, very recently in the year 2019, seven Federally Administered Tribal Areas (FATA) have been merged politically and administratively into the province of Khyber Pakhtunkhwa. The climate in this province is of the tropical monsoon type, but most of the districts are situated beyond the tropical zone with relatively high temperatures and a cool, dry winter that runs from December to February with March to June representing the hot and dry season. Summer extends from July to September and is generally rainy. October and November represent the receding monsoon period. The province has two rainy seasons: March to April and the summer monsoon from July to September.

### 2.2. Data Collection

The quarterly data on TB cases and population-at-risk in each district were collected from the quarterly reports (2015–2019) of the District Health Information System (DHIS) Khyber Pakhtunkhwa, Pakistan [39]. The District Health Information System (DHIS) collects data on the reported disease cases in each district on a monthly basis. All hospitals in a district report the registered disease cases to the respective DHIS office on a monthly basis. The district offices then send the monthly data to the provincial office, DHIS Cell, Khyber Pakhtunkhwa [40]. The collected dataset comprises the number of quarterly reported TB cases in each district and the corresponding population-at-risk. The collected dataset is available in supplementary information File S1.

### 2.3. Clusters Detection

The scan statistics software SaTScan™ ver.9.6 [41] with a discrete Poisson probability model was applied under retrospective analysis to detect the spatial and space-time clusters of TB cases at district-level in Khyber Pakhtunkhwa province, Pakistan from January 2015 to April 2019. SaTScan with the discrete Poisson model assumes the number of disease cases in each region is Poisson-distributed with the parameter *λ* as in Equation (1) [42].

(1) $$\lambda =\mu (z)\frac{nG}{\mu (G)}$$

where *µ*(*z*) denotes the population count for area *z*, *μ* (*G*) denotes the total population of the whole study area, and *nG* is the total observed disease counts in the whole study area. Under the null hypothesis of no cluster exists, the expected number of disease cases in each region is proportional to the population size in that region. SaTScan is an open source software program that was originally developed in [43]. The space-time scan statistics is defined by a cylindrical shaped window with a circular base. The base of the cylindrical window corresponds to the geographical size of the cluster and the height corresponds to the temporal length of the cluster. The circular base is centered around one of the several location-centroids in the study area, with the radius varying continuously from zero to the maximum spatial window size. The height of the cylinder is varying from zero to the maximum temporal window size. The cylindrical window is then moved in space as well as time to check the clusters with all possible spatial and temporal sizes. As a result, a number of overlapping cylinders of various sizes are obtained that jointly cover the entire study region. Each cylindrical window reflects a possible space-time cluster. The likelihood function is maximized over all cylindrical windows, and the window with the maximum log-likelihood ratio (LLR) is assumed to be the most likely cluster, that is, the cluster least likely to be caused by chance, and other windows with a statistically significant LLR were measured as secondary clusters. The statistical significance of each cluster in the study area is based on comparing the likelihood ratio (LLR) against a null distribution achieved from the Monte Carlo simulation. The detail of how SaTScan works is given in the SaTScan user guide [42]. 

In our study, the maximum spatial window size was set to the default value, i.e.,  ≤ 50% of the total population of the study area and the maximum temporal window size was set to default value, i.e. , ≤ 50% of the study period [42]. A circular window shape was chosen. A Monte-Carlo approach with 9999 repetitions was performed to test the null hypothesis that there was no difference in Relative Risk (RR) between the TB clusters. The clusters with *p*-value < 0.001 were considered as statistically significant clusters. 

## 3. Results and Discussion

**Figure 2:**
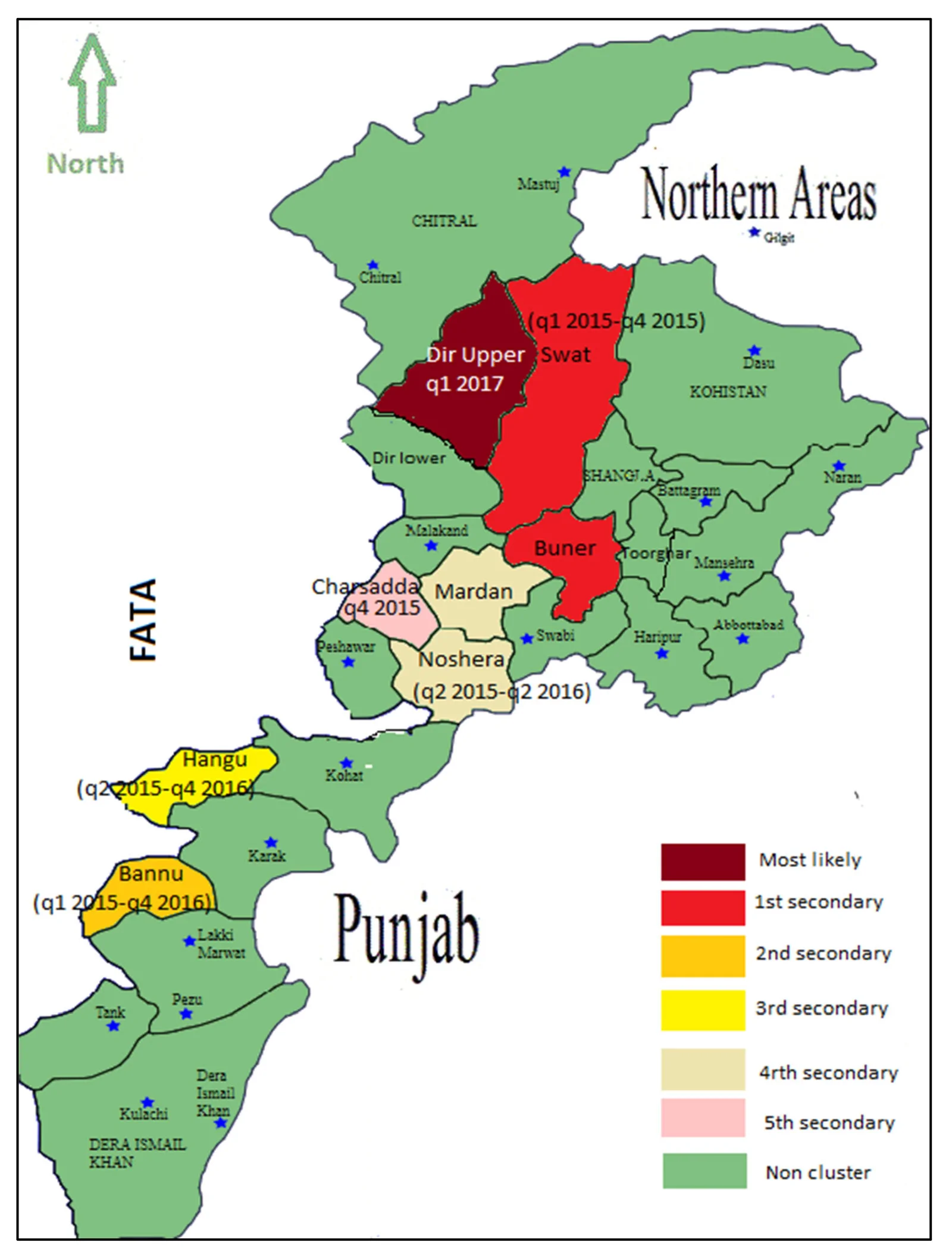
The locations of space-time clusters in the study area.

The space-time cluster analysis by SaTScan identified a total of six space-time clusters of TB cases in Khyber Pakhtunkhwa, Pakistan from 1st quarter 2015 to 1st quarter 2019 (Table 1, Figure 2). The most likely cluster was seen in the northern district Upper Dir for the 1st quarter of 2017. The 1st secondary cluster was seen in two northern districts Buner and Swat for the whole year 2015. The 2nd secondary cluster occurred in the western district Bannu for two years 2015–2016. The 3rd secondary cluster appeared in western district Hangu for 2nd quarter 2015 to 1st quarter 2016. The 4th cluster covered the two central districts Noshera and Mardan for the period of one year (from 2nd quarter 2015 to 2nd quarter 2016). All these clusters are statistically significant (*p*-value < 0.001). The results show that five of the potential clusters have occurred in the years 2015–2016, one in 2017, and none of the clusters was seen in the years 2018–2019. The interval 2015–2016 exhibits the largest number of clusters in the study period. The high TB incidence in the start of the study period (2015–2016) might be due to the lack of access to the healthcare because the number of healthcare centers in Khyber Pakhtunkhwa province was lower (786) in the year 2015 as compared to (1445) in 2018 [44]. 

The geographical locations of the detected space-time clusters of TB cases in the study area are shown in Figure 2, which indicates some important patterns of TB incidence in the study area. It is obvious from Figure 2 that in the 1st quarter 2015, two potential TB clusters occurred. The one covered the two districts (Swat and Buner) in the north persisted for one year (2015), which shows a tendency of spread toward the neighboring districts in the central part of the province from 2015 to 2016 and then moved again to the north (Upper Dir) in 2017. The other cluster in 1st quarter 2015 appeared in one district in the west persisted for two years (2015–2016), showing no tendency of spread toward the neighboring districts. Such a space-time pattern of the potential cluster provides clues to disease etiology and risk behaviors, suggesting local environmental or social characteristics that promote increased risk. Moreover, the retrospective clusters guide the public health officials on the significance of the control strategies that have been implemented previously in the cluster-regions to control the prevalence. 

To know the general pattern of spatial clusters of TB cases over time, we performed spatial cluster analysis for each year (2015–2018) using SaTScan. Five significant clusters were detected in the year 2015, seven in 2016, five in 2017 and four in 2018 (Table 2). The cluster with *p*-value < 0.001 is considered to be statistically significant. The largest number of spatial clusters were seen in 2016 compared to the other years. The interval 2015–2016 was also found to have a large number of clusters in space-time analysis (Table 1). 

**Figure 3:**
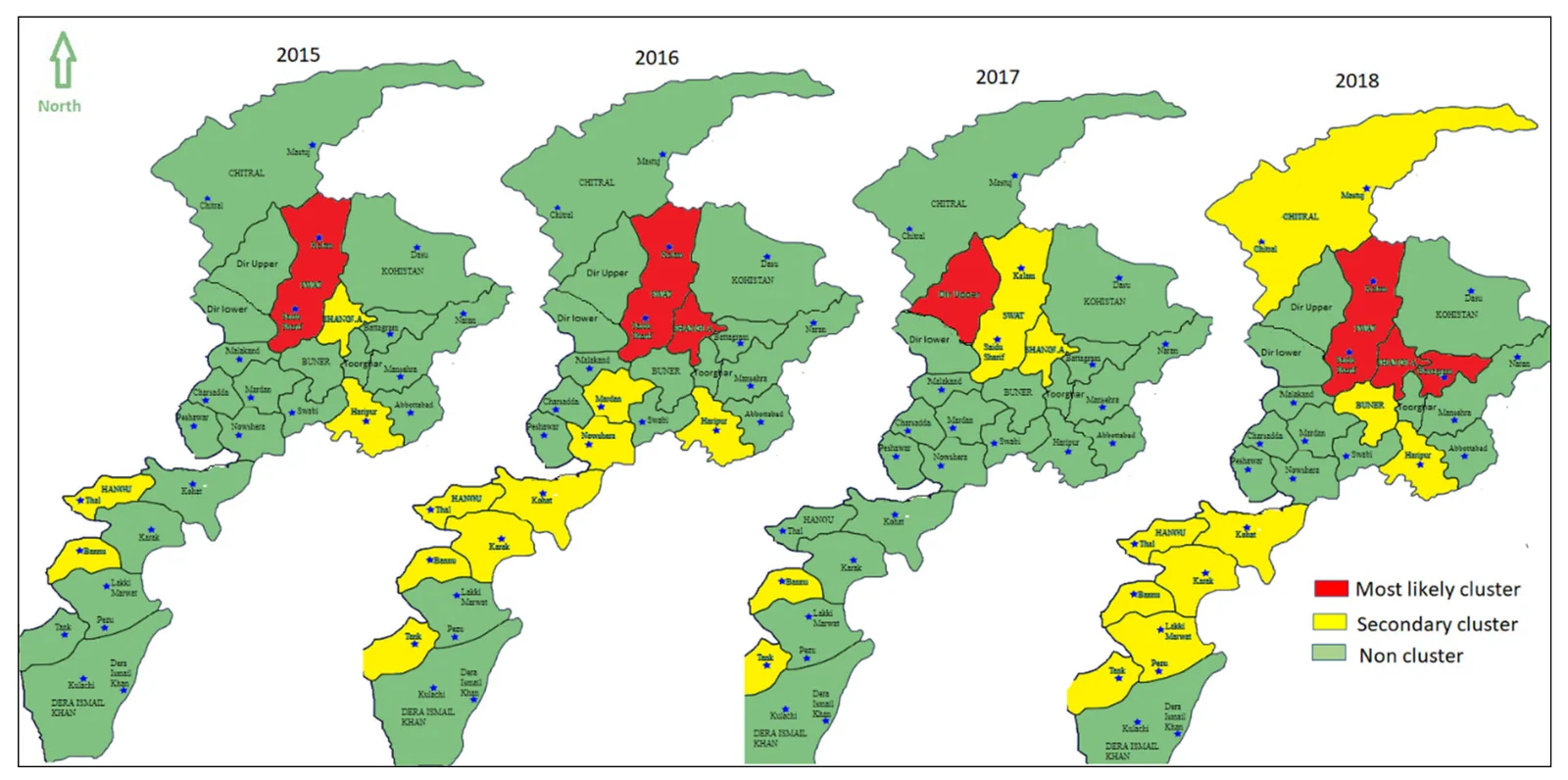
The locations of spatial clusters in the study area.

The geographical locations of the significant spatial clusters in each year were displayed in Figure 3. In 2015, the most likely cluster was seen in district Swat. In 2016, the same district with one neighboring district (Buner) appeared as the most likely cluster. In 2017, the most spatial cluster was moved from (Swat Buner) to the neighboring district Upper Dir in the west. The most likely cluster was moved back to districts Swat covering additional neighboring districts (Shangla and Battagram) in the South. The two districts (Swat and Buner) were found repeatedly in the potential cluster each year. The district Swat was seen in the most likely cluster in the year 2015, 2016, and 2018 showing it to be the main region of TB outbreak. Moreover, the two western districts Bannu and Hangu were found to be the spatial clusters throughout the study period (2016–2018). These results suggest three district Swat, Shangla, and Battagram for policymakers to be the most targeted districts for possible interventions because this part of the province was seen in the most likely cluster in each year (2016–2018). The two districts Bannu and Hangu are suggested to be the second targeted districts for possible interventions. These two secondary clusters show a tendency of spread toward the central districts in 2018. The TB case burden in the two western districts (Bannu and Hangu) might be due to the presence of the large proportion of Afghan refugees in these districts as evidence from the previous study [45]. Some previous studies on TB prevalence in the individual district have also been identified the evidence of TB outbreak in our resulted most likely cluster regions Swat and Buner [11,12].

It is evident from Figure 2 and Figure 3 that the most likely TB clusters occurred each year in rural hilly areas [46], which might be due to lack of access to healthcare. The population is scattered in these areas, i.e., the population is distributed in small villages that are very far from each other and hence most of the villages are very far from healthcare centers. In addition, the lack of awareness and misconceptions about TB might have caused TB clusters in these rural areas [47]. The snowfall and dry–cold climate in winter may also contribute to high TB cases in these districts.

## 4. Conclusions

This study provides a good understanding of the space-time clustering characteristics of TB in Khyber Pakhtunkhwa, Pakistan from 2015 to 2019. The most likely and the 1st secondary space-time clusters were seen in the northern part of the province such as (Dir Upper, Swat, and Buner), showing a tendency of spread toward the central part from 2015 to 2016 and then moved toward the north in 2017 (Figure 2). Most of these space-time clusters were seen in the years 2015–2016, which might be associated to the lack of healthcare centers in this period. In addition, the most likely spatial clusters were also seen each year in the northern districts (Dir Upper, Swat, and Shangla), showing a tendency of spread over the years from the north toward the neighboring districts in the south (Figure 3). This study suggests these northern districts for policymakers to be the most targeted regions for possible interventions. This targeted intervention may help control the TB epidemic in the province more effectively. Future research on finding the social and environmental determinants of TB outbreak in Khyber Pakhtunkhwa province is strongly recommended.

## Figures and Tables

**Table 1:** The detected space-time clusters of TB cases in Khyber Pakhtunkhwa, Pakistan.

Cluster Type	Districts	Number of Districts	Time-Frame	Observed Counts	Expected Counts	LLR	P-value
Most Likely	Dir Upper	1	1st quarter 2017	10686	219.37	31541.8	<0.001
1st secondary	Buner, Swat	2	1st quarter 2015 to 4th quarter 2015	13019	2599.46	11044.8	<0.001
2nd secondary	Bannu	1	1st quarter 2015 to 4th quarter 2016	4495	2001.46	1170.532	<0.001
3rd secondary	Hangu	1	2nd quarter 2015 to 4th quarter 2016	2216	822.69	810.864	<0.001
4th secondary	Noshera, Mardan	2	2nd quarter2015 to 2nd quarter 2016	6356	4203.86	496.057	<0.001
5th secondary	Charsadda	1	4th quarter 2015	505	365.45	23.865	<0.001

**Table 2:** The detected spatial clusters of TB cases in Khyber Pakhtunkhwa, Pakistan.

Year	Cluster Type	Districts	Number of Districts	Observed Counts	Expected Counts	LLR	P-value
2015	Most likely	Swat	1	12406	2500.81	11728.029	<0.001
	1st secondary	Bannu	1	2435	1292.42	20.132	<0.001
	2nd secondary	Hangu	1	1022	616.25	113.73	<0.001
	3rd secondary	Shangla	1	1102	852.78	34.255	<0.001
	4th secondary	Haripur	1	1381	1181.37	16.602	<0.001
2016	Most likely	Shangla, Swat	2	4794	2363.25	1097.165	<0.001
	1st secondary	Karak, Hangu, Bannu, Kohat	4	4814	2770.32	714.454	<0.001
	2nd secondary	Noshera	1	1891	1221.14	166.741	<0.001
	3rd secondary	Mansehra	1	1872	1350.35	95.685	<0.001
	4th secondary	Tank	1	568	332.69	69.656	<0.001
	5th secondary	Mardan	1	2371	2039.16	28.081	<0.001
	6th secondary	Haripur	1	1133	966.76	14.12	<0.001
	7th secondary	Dir Lower	1	1040	1002.26	0.731	0.997
2017	Most likely	Upper Dir	1	11025	957.11	18802.163	<0.001
	1st secondary	Bannu	1	2030	1181.08	262.797	<0.001
	2nd secondary	Swat	1	3053	2335.65	109.45	<0.001
	3rd secondary	Tank	1	573	396.31	35.082	<0.001
	4th secondary	Shangla	1	979	766.37	27.848	<0.001
	5th secondary	Hangu	1	599	524.66	5.125	0.065
	6th secondary	Haripur	1	1069	1014.36	1.496	0.859
2018	Most likely	Shangla, Swat, Battagram	3	7997	2672.79	4206.852	<0.001
	1st secondary	Bannu, Lakki Marwat, Karak, Hangu, Tank, Kohat	7	5796	3510.64	759.992	<0.001
	2nd secondary	Chitral	1	605	337.39	87.288	<0.001
	3rd secondary	Haripur	1	946	756.46	22.787	<0.001
	4th secondary	Mansehra	1	1266	1173.85	3.72	0.183
	5th secondary	Dir Lower	1	1153	1082.93	2.331263	0.570

## Supplementary Materials

The following are available online at https://www.mdpi.com/1660-4601/17/4/1413/s1, File S1: Data on TB cases and population-at-risk in Khyber Pakhtunkhwa, Pakistan (2015–2019).
